# Effect of slaughter age and breed on meat and kaddid quality – a comparative study of Noire de Thibar and Barbarine sheep breeds

**DOI:** 10.5194/aab-69-45-2026

**Published:** 2026-01-21

**Authors:** Amira Zioud, Martino Musati, Guido Mangione, Salvatore Gagliano, Wafa Hajji, Samir Smeti, Sihem Bellagha, Ines Essid

**Affiliations:** 1 National Agronomic Institute of Tunisia, Department of Food Technologies (UR17AGR01), University of Carthage, 43 Av. Charles Nicolle, Tunis 1082, Tunisia; 2 Department Di3A, University of Catania, via Santa Sofia 100, 95123 Catania, Italy; 3 Laboratoire des Productions Animales et Fourragères, INRA-Tunisia, University of Carthage, rue Hedi Karray, Ariana 2049, Tunisia

## Abstract

As demand for local quality products continues to grow, new ways of adding value to sheep meat are being explored. In this connection, cull ewe meat could be an interesting alternative to traditional products. This study aims to promote the use of cull ewes' meat in traditional meat products by evaluating the influence of age and breed on meat quality through a two-part approach. In the first part, the effects of age and breed were assessed on raw meat, while in the second part, the same parameters were investigated in traditional dried meat product “kaddid”. Two Tunisian sheep breeds, Noire de Thibar (NT) and Barbarine (BR), were examined at two age groups (3-month-old lambs and 5-year-old adults). Quality parameters, i.e. tocopherols, intramuscular fat (IMF) content, lipid peroxidation (TBARS), and fatty acid profile, were analysed for raw meat and kaddid samples. In raw meat, both breed and age factors significantly affected the α -tocopherol and total polyunsaturated fatty acid (PUFA) contents. Raw meat samples of BR breed showed higher levels of α -tocopherols and polyunsaturated fatty acids. However, no significant effects were revealed for γ -tocopherols, intramuscular fat, saturated fatty acids (SFAs), and lipid peroxidation levels. In kaddid samples, tocopherol content was affected by the slaughter age and revealed higher tocopherol content for ewes' kaddid samples ( P < 0.05), while intramuscular fat and TBARS showed no significant variation ( P > 0.05). Fatty acid groups were significantly affected by the age and breed factors, except SFAs, which were unaffected by age. The NT breed kaddid showed favourable levels of PUFA content, which can contribute to the recommended daily PUFA consumption for humans. Overall, both age and breed exerted clear effects on fresh meat and kaddid quality. In particular, meat from cull ewes showed advantageous tocopherol and PUFA profiles in dried products, highlighting its potential as a valuable raw material for traditional meat processing.

## 1 Introduction

The rich composition of meat and meat products in terms of protein, vitamins, and essential fatty acids makes this food a high-value nutritional source for human consumption. Lamb meat is highly appreciated in some Mediterranean countries for its characteristic flavour, aroma, and texture, which are closely related to its acceptance by consumers (Garmyn, 2020). Nevertheless, the physical and chemical composition of meat exposes it to several factors during the production and slaughter process, such as the oxidation process, spoilage by various chemical reactions, and microbial growth (Casaburi et al., 2015; Cunha et al., 2018). As a result, different techniques were developed to preserve and enhance the flavour of meat products. One of the most common processes is drying, which minimizes physical and chemical changes during meat storage (Chabbouh et al., 2011).

Dehydration removes water content in the meat, preventing the spread of microbial and biochemical reactions to preserve the quality and improve the meat's stability (Suput et al., 2019). Nowadays, dried meat, which is linked to local and traditional food models, has become a much-appreciated food product on the market because of its long shelf life, great taste, and health benefits (Mediani et al., 2022). Several varieties of traditional dried meats are produced worldwide, and one of these products is “kaddid”, produced in Maghreb countries, including Tunisia. This is a traditional cured lamb meat manufactured during the religious event “Aid”, whose production involves the meat's brine or dry-salting, spice addition, and sun-drying (Chabbouh et al., 2013).

Different studies reported how some ingredients' additions in kaddid preparation, such as salt or spices, and drying methods can affect the final feature of the product (Zioud et al., 2023; Chabbouh et al., 2013). However, there is no reported evidence on the impact of meat quality traits, associated with certain breeding factors, on the final characteristics of kaddid. Indeed, the animal's physiological state, post-mortem biochemistry, carcass composition, and diet, along with the effect of genetics on tissues and metabolism, may all represent determining factors for meat quality (Armstrong et al., 2018; Biondi et al., 2019). In addition, the age of the animal at slaughter, the sex, and the breed might greatly influence the nutritional characteristics of meat products (Cafferky et al., 2019; Klupsaite et al., 2022).

The breed is an important factor that can have a significant effect on the physicochemical characteristics of lamb meat (Martínez-Cerezo et al., 2005). Further to this, the use of local breeds can exert some advantages in meat production (Nikolaou et al., 2023). Indigenous sheep breeds are vital to contributing to the biodiversity of the production systems and to the conservation and sustainable use of livestock genetic resources that should be protected due to their climate adaptation, disease resistance, high fertility, and unique product qualities (Hiemstra et al., 2010; Mendelsohn, 2003). In Tunisia, ovine are among the major economically important livestock sector of the country, with about 6.5 million head, where the main meat breeds are represented by Barbarine (BB) and Noire de Thibar (NT), better adapted to harsh environmental conditions (Salem et al., 2011). Several research articles have been published on these two indigenous meat breeds, focusing on all aspects concerning feeding, reproduction, management, and genetics (Djemali et al., 2021; Hajji et al., 2016; Atti and Mahouachi, 2011).

In sheep meat production, one category of the flock (until 40 %) is represented by cull ewes, which are females over 1 year old that are no longer needed for breeding and are destined for slaughter (Vasconcelos-Filhoa et al., 2021). This sheep category is usually poorly appreciated, due to the lowest body condition, live weight, and carcass yield (Fruet et al., 2016; Bhatt et al., 2012). Organoleptically different from sheep meat, the raw meat of cull ewes is generally considered undesirable and has limited market and low economic value. For these reasons it is commonly used for the production of processed meat products through a drying and salting process, increasing its commercial value (Andrade et al., 2017).

Reports of many studies on lamb and cull ewe meat quality and characteristics were conducted (Abdelmalek et al., 2020; Panea et al., 2023). However, no study was found on using ewe meat in kaddid production.

Therefore, in order to identify and validate a way to promote cull ewes' meat, the present work was conducted to study the effect of the slaughtering age (3-month-old lambs and 5-year-old cull ewes) and breeds (Barbarine and Noire de Thibar) on the oxidation rate and fatty acid profile of a dried meat product, kaddid. Indeed, this would allow the marketing of cull ewe meat as a traditional ethnic product and ensure the maintenance and sustainability of the sheep value chain in Tunisia.

## 2 Materials and methods

### 2.1 Sample preparation

This study was conducted in the governorate of Bizerte in North Tunisia, where all the animals were reared on commercial breeding farms. Meat samples were taken from two local breeds, Barbarine and Noire de Thibar. Two age groups were selected (3-month-old lambs and 5-year-old ewes) within each breed. Male lambs and ewes chosen in the present study were intended to represent the same production system and were reared under the same feeding system. Ewes were fed oat hay ad libitum and 750 g of commercial concentrate (per ewe and day), and lambs received oat hay ad libitum supplemented with 500 g of the same commercial concentrate. The concentrate was composed of barley (80 %), soybean meal (18 %), and mineral vitamin supplement (2 %).

Animals were slaughtered on the same day in September 2022 at a commercial abattoir. After cooling at 4 °C for 24 h, meat samples were collected from the leg muscles. One portion of the raw meat was stored at - 20 °C until analysis of fatty acid composition, tocopherol content, and lipid peroxidation. The remaining portion was used for the preparation of kaddid samples, following the processing diagram described by Zioud et al. (2023). Meat samples were cut into strips and then seasoned with a powdered spice mixture of garlic powder (4 g/100 g of meat), coriander powder (6 g/100 g of meat), salt (1.5 g/100 g of meat), and paprika (0.5 g/100 g of meat). After seasoning, the strips of meat were dried in an oven (SHEL LAB model 1375 FX, USA) at a constant air temperature of 35 °C and an airflow velocity of 3 m s^−1^ for 45 h. The selection of this drying method was justified by the findings reported by Zioud et al. (2023). The resulting kaddid samples were stored at - 20 °C until analysis.

### 2.2 Chemical analyses

#### 2.2.1 Tocopherols

Tocopherol contents of raw meat and kaddid were determined following the methodology described by Scerra et al. (2022) and modified by Menci et al. (2023). In brief, 0.5 g of minced sample was saponified in dark overnight with 200 mg of ascorbic acid and 7.5 mL of saponify solution (10 % w:v KOH, 50 : 50 v:v EtOH : H_2_O) and shaken at 22 °C (250 rpm) under a nitrogenous atmosphere. Subsequently, the analytes were extracted twice with 5 mL of hexane : ethyl acetate 9:1 v:v (with 25 µ g mL^−1^ BHT) and centrifuged at 2000×g for 5 min at 10 °C. The evaporation of the supernatant was carried out under nitrogen flow at 40 °C. Subsequently, 1 mL of methanol was added to the pellet then filtered through a PTFE syringe filter (0.2 µ m/13 mm) into a 2 mL vial. Quantification of tocopherols was carried out using a Nexera UHPLC (Shimadzu Corporation, Kyoto, Japan) equipped with a C18 phase column (Zorbax ODS, Supelco, Bellefonte, PA; length: 25 cm; internal diameter: 4.6 mm; particle size: 5 µ m). Tocopherols were detected by measuring the fluorescent emission at λ exc = 295 nm and λ emi = 330 nm. The comparison with the retention time of pure standards (Merck Life Science s.r.l., Milan, Italy) was used to identify the analytes, and for each of them, external calibration curves were created with pure standards.

#### 2.2.2 Intramuscular fat and fatty acid profile

The intramuscular fat (IMF) of raw meat and kaddid samples was extracted according to the method of Folch et al. (1957). Fatty acid methyl esters (FAMEs) were subsequently prepared following the transesterification procedure described by Christie (1982), with minor modifications. Sodium methylate in methanol (0.5 M CH_3_ONa) was used as a basic catalyst, and nonadecanoic acid (C19:0) was employed as the internal standard (1 mg mL^−1^). Briefly, 2 mL of total lipid extract (hexane : 2-propanol, 4 : 1, v/v ) was transferred into pre-weighed rotary evaporator tubes, and the solvent was evaporated at 30 °C under vacuum. The residue was kept in the dark overnight. On the following day, 1 mL of sodium methylate solution was added, vortexed for 3 min, and allowed to stand for 5 min. Subsequently, 2 mL of the internal standard solution was added, and the upper hexane phase (1–1.5 mL) was collected for analysis.

FAMEs were separated and quantified using gas chromatography (Thermo Finnigan Trace GC, ThermoQuest, Milan, Italy) equipped with a flame ionization detector and a highly polar fused silica capillary column (SP-2560, 100 m × 0.25 mm × 0.25 µ m). Identification was performed by comparing retention times with authentic reference standards. Quantification was performed using the area normalization method, and the relative proportion of each fatty acid was expressed as a percentage of the total identified FAME. Instrument calibration was verified daily using a standard mixture to ensure reproducibility and accuracy of retention times and peak areas.

#### 2.2.3 Lipid peroxidation

Lipid peroxidation of meat and kaddid samples was measured at day 0 following the method of thiobarbituric acid reactive substances (TBARSs) described by Natalello et al. (2020). Briefly, 2.5 g of minced samples was mixed in 12.5 mL of distilled water and homogenized for 2 min at 9500 rpm (Ultra Turrax T-18 Homogenizer), keeping the samples in an ice water bath. Subsequently, 12.5 mL of 10 % ( w/v ) trichloroacetic acid (TCA) was added and mixed thoroughly with the sample. After filtering the supernatant (Whatman filter No. 1), 4 mL of the filtrate was mixed with 1 mL of 0.06 M thiobarbituric acid (TBA) and incubated in a water bath at 80 °C for 90 min. The absorbance was measured at 532 nm using a UV–vis spectrophotometer (UV-1601; Shimadzu Corporation, Milan, Italy). A calibration curve was prepared using 1,1,3,3-tetraethoxypropane (TEP) as a standard precursor of malondialdehyde (MDA). The MDA concentration of each sample was calculated from the standard curve, and results were expressed as mg MDA kg^−1^ of meat.

### 2.3 Statistical analyses

All the measurements were conducted in triplicate. The statistical analysis of the results obtained was performed using the software IBM SPSS version 23. A two-way analysis of variance (ANOVA) was carried out for the animal's age at slaughter ( A ) and breed ( B ) and for the interaction between breed and age ( A⋅B ). The difference was considered significant at P < 0.05. Principal component analysis (PCA) was performed on the data set to be sure whether it was possible to differentiate the samples according to their breed and age at slaughter.

## 3 Results and discussion

### 3.1 Meat characterization

#### 3.1.1 Chemical composition

The results of tocopherol composition and intramuscular fat (IMF) of raw meat samples are summarized in Table 1. Both age ( P < 0.05) and breed ( P < 0.01) had significant effects on the α -tocopherol composition of raw meat samples. Values obtained from Age 2 (5 years old) were greater compared to values from Age 1 (3 months old) (9.13 µ g g^−1^ dry matter (DM) vs. 4.15 µ g g^−1^ DM and 7.15 µ g g^−1^ DM vs. 3.06 µ g g^−1^ DM, respectively). These results were consistent with those found by other authors comparing the nutritional quality of female and male lambs' meat with ewes' meat, reporting higher value of α -tocopherol content in ewes' meat samples (Panea et al., 2023). Similar results were also reported by Cougo et al. (2024), revealing higher α -tocopherol concentrations in 12-month-old lambs compared to 4- and 6-month-old lambs. Since these compounds are fat-soluble, they are deposited in the IMF, which was higher than 2-fold in Age 2 compared to Age 1 (0.90 g kg^−1^ fresh matter (FM) vs. 0.41 g kg^−1^ FM and 0.52 g kg^−1^ FM vs. 0.40 g kg^−1^ FM, respectively), confirming what was already reported in the literature by Panea et al. (2023). With regard to the breed effect, samples from the BR breed showed greater range values of α -tocopherol content than those from the NT breed. The concentrations measured in BR lamb meat in the present study exceeded those reported for BR control samples by Yagoubi et al. (2021) and Tibaoui et al. (2020). This superiority is likely attributable to environmental and nutritional factors, as α -tocopherol levels in tissues are highly sensitive to dietary intake. The inter-breed variation was in agreement with Ponnampalam et al. (2021), who reported a significant difference in α -tocopherol concentrations between raw meat samples of two Australian lamb breeds. Neither age nor breed showed a significant effect on γ -tocopherols, which is in agreement with the findings of. (Panea et al., 2023). However, their γ -tocopherol contents measured for ewes and lambs from the Merino breed were higher in comparison with the present study. This difference in vitamin E levels can be explained by the different rearing conditions and grazing systems. In fact, Ponnampalam et al. reported a significant difference in vitamin E levels in lamb meat derived from two grazing systems (Ponnampalam, 2012). According to Díaz et al. (2010) and Domínguez et al. (2019), tocopherol values between 30 and 80 µ g g^−1^ DM in dried or cured meat products are considered optimal for maintaining oxidative stability and contributing to the recommended vitamin E intake.

**Table 1:** Tocopherol and intramuscular fat (IMF) content of raw meat.

Item	BR		NT			P value		
	Age 1	Age 2	Age 1	Age 2	SEM	Age ( A )	Breed ( B )	A⋅B
α -Tocopherols ( µ g g^−1^ DM)	4.15^b^	9.13^d^	3.06^a^	7.15^c^	0.74	^*^	^**^	NS
γ -Tocopherols ( µ g g^−1^ DM)	0.31	0.19	0.18	0.3	0.02	NS	NS	^**^
IMF (g kg^−1^ FM)	0.41	0.90	0.40	0.52	0.07	NS	NS	NS

BR: Barbarine breed; NT: Noire de Thibar breed; Age 1: 3 months; Age 2: 5 years; DM: dry matter; FM: fresh matter; NS: not significant; ^*^ p < 0.05; ^**^ p < 0.01; ^***^ p < 0.001; A⋅B : interaction between age and breed; a, b, c, d: different letters within the same row differ significantly ( P < 0.05).

The statistical analysis showed no significant effect of breed on IMF content, probably because the two breeds were reared in the same conditions. In other research characterizing the NT and BR breed meat, the IMF was affected by breed with the leaner meat for NT compared to BR and showed significant interaction between breed and feeding system (Hajji et al., 2016, 2014). The IMF content represents an important parameter affecting the nutritional value and the sensory traits of meat, particularly juiciness, flavour, and tenderness (Cadavez et al., 2020). Several studies attribute the difference in IMF to the grazing system: Cougo et al. (2024) reported a lower proportion of IMF for lambs fed mixed pastures compared to lambs grazed in a pastoral sheep intensive unit. Similarly, Popova (2007) reported greater intramuscular lipid content in intensively reared lambs than in extensively reared lambs. Vermorel (1988) suggested that the decrease in lipid deposition in extensively reared animals could be attributed to the higher energy spent on locomotion and thermoregulation. There was no significant effect or interaction observed between ages and breeds on the intramuscular fat content of meat samples ( P > 0.05). The absence of any effect of the slaughter age on IMF results was in agreement with the research reported by Pavan et al. (2022) on the East Friesian crossbred lamb meat. However, they found higher values than those obtained in this study.

#### 3.1.2 Fatty acid profile

The evaluation of the fatty acid (FA) profile of raw meat samples is reported in Table 2. Generally, the FA proportion of meat is mainly influenced by the production system and the feeding management of the animals, with high levels of saturated FAs (SFAs) (Cadavez et al., 2020).

**Table 2:** Fatty acids and fatty acid groups ( % of total FAME) of raw meat.

Item	BR		NT			P value		
	Age 1	Age 2	Age 1	Age 2	SEM	Age ( A )	Breed ( B )	A⋅B
∑ SFA	45.5^c^	42.5^a^	43.3^ab^	44.3^bc^	0.40	NS	NS	^**^
C12:0	0.28^b^	0.22^a^	0.27^b^	0.31^c^	0.01	NS	^**^	^***^
C14:0	2.60^b^	2.38^ab^	2.26^a^	3.20^c^	0.12	^**^	^*^	^***^
C16:0	20.6^a^	21.0^ab^	21.2^ab^	21.6^b^	0.15	NS	^*^	NS
C17:0	1.46^c^	1.17^ab^	1.35^b^	1.14^a^	0.04	^**^	^*^	NS
C18:0	17.8^b^	15.3^a^	16.5^a^	15.2^a^	0.37	^**^	NS	NS
C20:0	0.12	0.12	0.12	0.13	0.002	NS	NS	NS
∑ MUFA	42.3^a^	41.4^a^	44.9^b^	41.3^a^	0.5	NS	^**^	^*^
C16:1 *cis*-9	1.25^a^	1.36^b^	1.27^a^	1.42^b^	0.02	^***^	NS	NS
C18:1 *cis*11	1.54^b^	1.55^b^	1.45^ab^	1.36^a^	0.03	NS	^**^	NS
C18:1 *cis*-9	32.9	32.6	32.7	31.6	0.29	NS	NS	NS
∑ PUFA	12.2^a^	16.1^c^	11.8^a^	14.5^b^	0.53	^***^	^***^	^**^
C18:2 n-6	6.70^a^	9.20^c^	7.57^b^	7.91^b^	0.28	^***^	NS	^***^
CLA	0.78^c^	0.56^a^	0.8^c^	0.71^b^	0.03	^***^	^**^	NS
C18:3 n-3	2.18^b^	2.19^b^	1.43^a^	2.70^c^	0.14	^***^	^*^	^***^
C20:4 n-6	1.66^a^	2.53^c^	1.48^a^	2.12^b^	0.13	^***^	^**^	NS
PUFA / SFA	0.27^a^	0.38^c^	0.27^a^	0.33^c^	0.01	^**^	^***^	^**^

^*^BR: Barbarine breed; NT: Noire de Thibar breed; Age 1: 3 months; Age 2: 5 years; A⋅B : interaction between age and breed; DM: dry matter; FM: fresh matter; MUFA: monounsaturated fatty acid; PUFA: polyunsaturated fatty acid; CLA: conjugated linoleic acid; NS: not significant; ^*^ p < 0.05; ^**^ p < 0.01; ^***^ p < 0.001; a, b, c, d: different letters within the same row differ significantly ( P < 0.05).

In this study, all the meat samples from each group showed a high proportion of SFAs (42.5 % and 45.5 % in BR Age 2 and 1, respectively) and monounsaturated FAs (MUFAs) (41.3 %–44.9 % in NT Age 2 and 1, respectively), followed by polyunsaturated FAs (PUFAs) (11.8 %–16.1 % in NT Age 1 and BR Age 2, respectively). As a comparison, Gonzales-Barron et al. (2021) reported higher SFA content and a lower proportion of MUFAs in lamb meat of three Italian and German local breeds, i.e. Sambucana, Biellese, and crossbred Texel–Merino–Blackhead–Charollais. It should be noted, however, that only Sambucana lamb presented higher PUFA content (20.4 %) than other studied meat samples. In the present study, no differences were observed in total SFA content, with the higher value reported by Age 1 BR samples than by the others, in line with Hajji et al. (2016). However, MUFA and PUFA contents were affected by the breed, and BR had a higher proportion of PUFA than NT. Conversely, Hajji et al. (2016) reported no significant effect between BR and NT breeds on the same FA groups.

Concerning individual fatty acids, the main SFAs were palmitic acid (C16:0), ranging from 20.6 % to 21.6 % of total FA, and stearic acid (C18:0), ranging from 15.2 % to 17.8 % of total detected FAs. These results aligned with values reported by other studies conducted on lamb intramuscular FAs (Cadavez et al., 2020; Bas et al., 2007). From a health point of view, the C16:0 and C18:0 increased dietary intake is commonly associated with a higher risk of coronary heart disease (Zong et al., 2016). Moreover, the results showed that C12:0, C14:0, C16:0, and C17:0 were all affected by breed, and C14:0 and C17:0 were also affected by age. The NT group had a lower percentage of C16:0 than BR; however, the opposite was observed for the C18:0 content. Interestingly, the effect of breeds on the FA content of meat has been well highlighted in several studies focusing on different sheep breeds (Gonzales-Barron et al., 2021; Hajji et al., 2016).

The major MUFA for all groups was oleic acid (C18:1 c 9), which counted around 76 % of total MUFAs and 32 % of total FAs. This predominant monounsaturated fatty acid showed no significant differences among ages and breeds. It was reported that C18:1 c 9 has beneficial effects against cancer and inflammatory diseases; thus its higher proportion in lamb meat may exert useful influence on human health (Cadavez et al. 2020; Sales-Campos et al., 2013).

Significant differences ( P < 0.001) were observed among breeds and slaughter ages in total PUFA contents. The BR breed presented a higher proportion of PUFAs compared to NT at both ages evaluated, while Age 2 showed higher PUFA levels than Age 1 in both breeds (Table 2). Among the individual fatty acids, linoleic acid (C18:2 n-6) was predominant in all samples, followed by α -linolenic acid (C18:3 n-3). Linoleic acid, the primary dietary n-6 PUFA, may confer cardiometabolic benefits rather than harmful effects, although maintaining a balanced n-6 / n-3 ratio remains important for optimal health (Ryman et al., 2017; Visioli and Poli, 2025).

Moreover, the high proportion of SFAs in the lamb meat may be associated with low values of the PUFA / SFA ratio, showing significant differences among ages and breeds and their interaction. However, the results on the PUFA / SFA ratio in all the meat samples were lower than the recommended values of 0.4 in the meat, ranging from 0.27 to 0.38 (Gonzales-Barron et al., 2021).

### 3.2 Kaddid characterization

#### 3.2.1 Chemical composition

The tocopherol composition and intramuscular fat (IMF) of kaddid are presented in Table 3. The age effect on α -tocopherol levels was significant for the NT breed ( P < 0.005), indicating a significant change between Age 1 and Age 2 (0.92 vs. 2.45 µ g g^−1^ DM). However, for the BR breed, the difference was not statistically significant ( P > 0.05), suggesting less variation in α -tocopherol levels with age compared to the NT breed. A significant ( P < 0.01) interaction between age and breed ( A⋅B ) was also observed, suggesting that the effect of age on α -tocopherol levels is different across breeds. Statistical analyses showed a consistent effect of age on γ -tocopherol levels. The γ -tocopherol content was higher for the BR breed kaddid compared to the NT breed kaddid at both Age 1 (0.26 vs. 0.12 µ g g^−1^ DM) and Age 2 (0.27 vs. 0.24 µ g g^−1^ DM). Similar tocopherol values were reported in a previous study in which the effects of different drying methods and storage time on kaddid samples were evaluated (Zioud et al., 2023).

**Table 3:** Tocopherol content and intramuscular fat (IMF) of kaddid.

Item	BR		NT			P value		
	Age 1	Age 2	Age 1	Age 2	SEM	Age ( A )	Breed ( B )	A⋅B
α -Tocopherols ( µ g g^−1^ DM)	1.91^b^	2.07^b^	0.92^a^	2.45^b^	0.19	^**^	NS	**
γ -Tocopherols ( µ g g^−1^ DM)	0.26^b^	0.27^b^	0.12^a^	0.24^b^	0.02	^*^	NS	^*^
IMF (g kg^−1^ FM)	1.11	2.63	1.27	0.98	0.26	NS	NS	NS

^*^BR: Barbarine breed; NT: Noire de Thibar breed; Age 1: 3 months; Age 2: 5 years; DM: dry matter; FM: fresh matter; A⋅B : interaction between age and breed; NS: not significant; ^*^ p < 0.05; ^**^ p < 0.01; ^***^ p < 0.001; a, b, c, d: different letters within the same row differ significantly ( P < 0.05).

There were no significant differences in IMF content between the ages and breeds and their interaction ( P > 0.05). This result reflected the absence of the effect of these two factors on raw meat used for kaddid preparation, as reported in the above section. It is worth noting that research studies dealing with the effect of breeds and age on the characterization of processed meat products are limited and even rare when it comes to a dried meat product such as kaddid.

#### 3.2.2 Fatty acid profile

The results of fatty acid content and fatty acid groups of kaddid samples are included in Table 4. The data obtained showed a significant effect of age and breed on all groups of fatty acids, except for SFA, for which no significant variation was observed as a function of age.

**Table 4:** Individual and groups of fatty acids (% of total FAME) of kaddid.

	BR		NT			P value		
Item	Age 1	Age 2	Age 1	Age 2	SEM	Age ( A )	Breed ( B )	A⋅B
SFA	45.0^b^	46.0^bc^	41.4^a^	47.7^c^	0.74	NS	^***^	^**^
C12:0	0.10^b^	0.32^c^	0.07^a^	0.41^d^	0.04	^***^	^*^	^***^
C14:0	2.20^b^	2.33^b^	1.60^a^	3.97^c^	0.27	^***^	^***^	^***^
C16:0	23.1^b^	23.7^b^	18.5^a^	22.3^b^	0.65	^**^	^***^	^*^
C17:0	1.47^d^	0.92^a^	1.10^b^	1.28^c^	0.06	^***^	NS	^***^
C18:0	16.0^a^	17.1^ab^	18.0^b^	16.7^ab^	0.29	NS	NS	*
C20:0	0.11^ab^	0.11^a^	0.12^b^	0.12^b^	0.002	NS	^*^	NS
MUFA	44.5^b^	45.1^b^	45.8^b^	40.1^a^	0.73	^*^	^**^	^**^
C16:1 *cis*-9	1.79^c^	1.41^b^	1.18^a^	1.45^b^	0.07	NS	^***^	^***^
C18:1 *cis*-11	1.76^c^	1.40^b^	1.54^b^	1.10^a^	0.07	^***^	^***^	NS
C18:1 *cis*-9	35.8^bc^	36.2^c^	33.8^ab^	31.7^a^	0.61	NS	^**^	NS
PUFA	10.5^b^	9.00^a^	12.7^d^	12.2^c^	0.45	^***^	^***^	^*^
C18:2 n6	6.68^b^	6.01^a^	7.17^b^	6.50^a^	0.15	^*^	^*^	NS
CLA	0.45^a^	0.43^a^	0.95^c^	0.74^b^	0.07	^***^	^***^	^***^
C18:3 n3	1.45^a^	1.31^a^	1.61^b^	2.31^c^	0.12	^***^	^***^	^***^
C20:4 n6	1.25	0.94	2.10	1.79	0.14	^***^	^***^	NS
PUFA / SFA	0.23^b^	0.20^a^	0.31^d^	0.26^c^	0.01	^***^	^***^	NS

^*^BR: Barbarine breed; NT: Noire de Thibar breed; Age 1: 3 months; Age 2: 5 years; A⋅B : interaction between age and breed; DM: dry matter; FM: fresh matter; MUFA: monounsaturated fatty acid; PUFA: polyunsaturated fatty acid; CLA: conjugated linoleic acid; NS: not significant; ^*^ p < 0.05; ^**^ p < 0.01; ^***^ p < 0.001; a, b, c, d: different letters within the same row differ significantly ( P < 0.05).

For both investigated breeds, the total amount of SFA was higher in samples of Age 2 compared to Age 1, with the highest SFA content for Age 2 NT breed samples (47.7 %). The SFA content found was in line with that previously reported by Zioud et al. (2023) on sun-dried kaddid samples and similar to the SFA content of salted and ripened goat legs (Paleari et al., 2008) and cured sheep legs (Teixeira et al., 2017).

The levels of MUFA in kaddid were influenced by age and breed and their interaction. In particular, the highest and lowest MUFA content were measured for NT samples at Age 1 (45.8 %) and Age 2 (40.1 %), respectively. Similar MUFA results were also reported by Teixeira et al. (2017) on cured sheep legs.

In addition, the analyses of FA content revealed a significant effect of breed and age and their interaction on the PUFA content and a significant effect of breed and age on the ratio PUFA / SFA. The NT samples presented the highest PUFA and PUFA / SFA ratio values (ranging from 12.1 to 12.2 and from 0.31 to 0.26, respectively) compared to BR samples at the two ages. Comparing the lamb meat kaddid (Age 1) with the ewes' meat product (Age 2), a slight tendency for a reduction in PUFA content with age was observed and thus evidenced differences linked to the types of animals investigated (Teixeira et al., 2017). The current results revealed a higher PUFA content and PUFA / SFA ratio for all investigated groups than those recently presented by Lopez et al. (2024) when characterizing two dry-salted products from the Italian Bergamasca sheep breed. In particular, the ratio PUFA / SFA is widely used to evaluate the nutritional value of fat in the human diet and to make food recommendations. The PUFA / SFA found in all kaddid samples was within the recommended limits and, according to Wood et al. (2008), within the ratio guidelines recommended above 0.4–0.5. This is generally considered favourable for health, as a high ratio is often emphasized as part of a heart-healthy diet. Similar ratio values were also reported by Teixeira et al. (2017) on cured sheep legs.

For fatty acids, age effect influenced six of the nine fatty acids presented. Similar results were also described by other researchers for barren Merino ewes (Panea et al., 2023). The higher proportion of conjugated linoleic acid observed in NT compared with BR may confer a nutritional advantage for human health. The C18:2 c9t11 isomer, the predominant form of CLA in ruminant fats, has been associated with beneficial effects on cardiovascular health, obesity, and cancer (Benjamin et al., 2015). However, both major CLA isomers (c9t11 and t10c12) have been linked to insulin resistance in humans, and adverse effects, including hepatic steatosis, have also been reported (Cordoba-Chacon et al., 2019). Consequently, despite the potential benefits of CLA and linoleic acid, careful consideration of isomer type, intake level, and overall dietary context is warranted.

### 3.3 Principal component analyses

**Figure 1:**
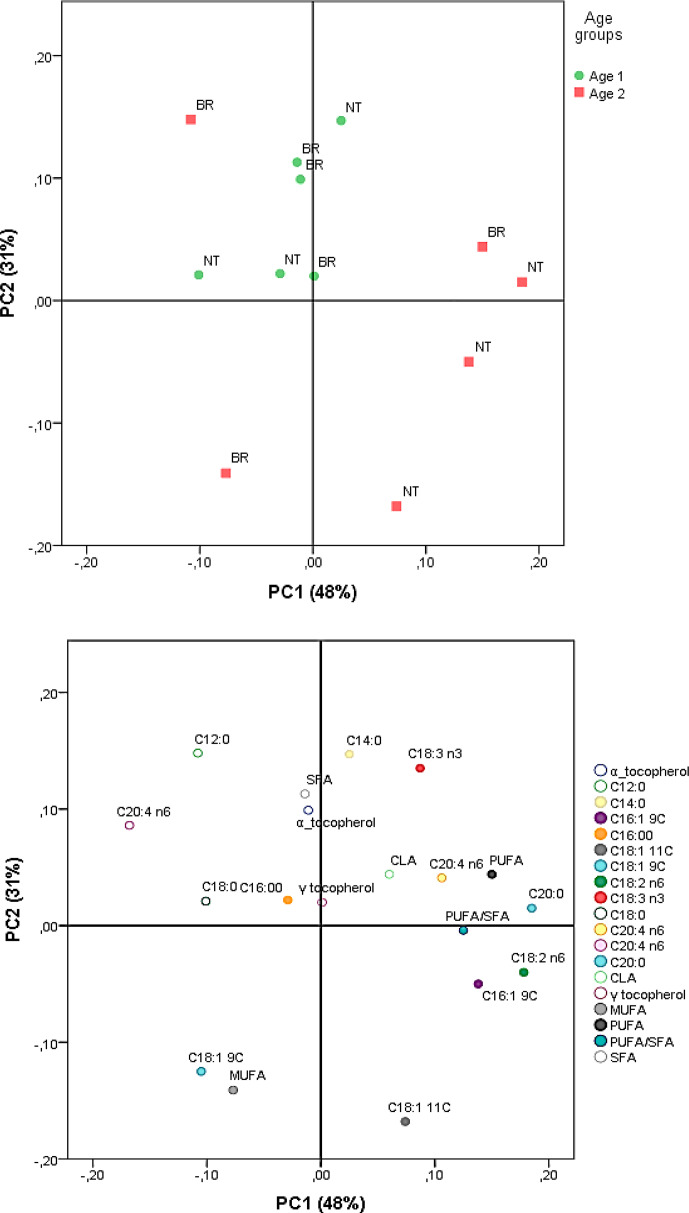
PCA score and loading plots.

Principal component analyses were carried out on the parameters measured for the kaddid samples to investigate the primary sources of variability and the importance of the variables measured. The first two PCs (PC1 and PC2) explained 48 % and 31 % of the total variance, respectively, accounting together for 79 % of the overall variability (Fig. 1). The score plot presented illustrated a distinct separation of samples, mainly based on slaughter age and, less significantly but still noticeably, based on breed. PC1 was positively correlated with C20:0, C18:2 n-6, C16:1 9C, C18:1 11C, and total PUFA, suggesting that samples from older animals tended to have higher levels of PUFA and MUFA, reflecting age-related changes in lipid metabolism.

Conversely, variation in PC2 had less pronounced determinants but included effects from variables such as C12:0, C14:0, and C18:3 n3. The BR breed samples clustered towards higher values of PUFAs and C18:2 n-6, while the NT samples were characterized by relatively higher levels of SFAs. Negative values for some variables suggested a potential inverse relationship between them and others that positively influence this component. Overall, the PCA results highlighted the significant impact of slaughter age and breed on the fatty acid and tocopherol composition of the kaddid samples and demonstrated the combined influence of genetic and physiological factors on the lipid quality of this traditional dried meat product.

### 3.4 Lipid peroxidation

**Figure 2:**
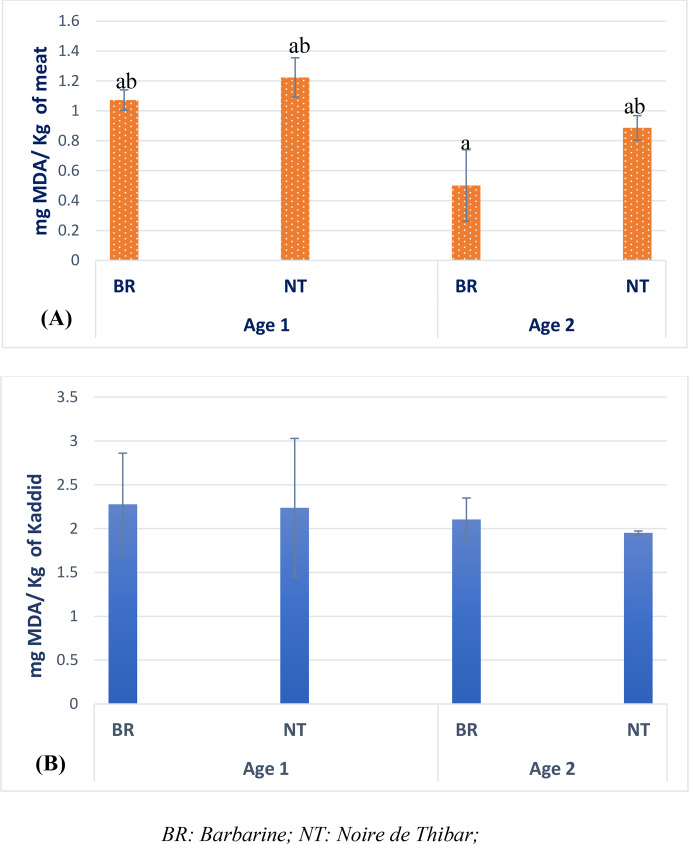
Lipid peroxidation of raw meat **(A)** and kaddid **(B)** samples.

Lipid peroxidation, along with microbial growth, is one of the primary mechanisms leading to the deterioration of food quality (Barden and Decker, 2016, Xavier et al., 2016). Figure 2 presents the lipid peroxidation of cull ewes' and lambs' raw meat and kaddid samples for both breeds. As expected, the obtained TBARS values showed higher levels of lipid peroxidation in kaddid than in raw meat samples. During the processing of dried food products, lipid peroxidation may be the main cause of significant changes in colour, aroma, and nutritional content (Barden and Decker, 2016; Wang et al., 2023). The levels of TBARS in dried meat products, such as kaddid, are higher because of their preparation process, i.e. mixing and drying, giving rise to significant deterioration of the cellular structures which allow the meat and pro-oxidants to fuse together (Mishra et al., 2017). Moreover, the lower TBARS level in raw meat compared to the kaddid samples may be also ascribed to the higher amount of α -tocopherol highlighted in raw meat, which is commonly known for its antioxidant capacity (Lopez et al., 2001). Therefore, the large increase in TBARS in the kaddid samples is in agreement with the lowered content of tocopherol.

Concerning the effects of breed and age, no significant evidence was revealed. All the TBARS values presented in the present study ranged between 1 and 2 mg MDA kg^−1^ of sample, corresponding to the range reported for consumer acceptance in meat (Campo et al., 2006; Gobert et al., 2010). The obtained results were favourable when compared with the oxidation level reported for salted and dried cull ewe meat by Oliveira et al. (2014), Dorg et al. (2015), respectively (2.16 and 2.59 mg MDA kg^−1^ of sample), and similar to those reported by Teixeira et al. (2017) in cured sheep samples.

Given all the above aspects reported in the present study, the use of cull animals for the production of the traditional kaddid might represent a reliable base for the estimation of its quality and therefore give an economic sense to promote the use of these animals for producing processed dry meat products. The marketing of cull ewes could then become an additional source of income for producers, representing an important culinary market segment, especially in the semi-arid regions (Abdelmalek et al., 2019).

## 4 Conclusion

The present findings demonstrated that slaughter age and breed had no significant effects on the lipid peroxidation levels and the intramuscular fat content of meat and kaddid derived from Tunisian sheep. However, higher tocopherol levels in products from aged ewes (Age 2) suggest improved oxidative stability in the final processed product. Although no variation in SFA content was observed, NT kaddid samples showed favourable levels of PUFA content compared to BR samples.

These results highlight the potential use of cull ewes, particularly from the Noire de Thibar breed, for the production of nutritionally valuable dried meat products. Such findings may guide local breeding programmes toward selecting animals with superior nutritional traits, promote the commercial valorization of cull animals in value-added products, offer the opportunity to enhance the economic efficiency of sheep production systems, and contribute to establishing substantiated nutritional claims for traditional foods.

## Data Availability

Data are available from the corresponding author upon reasonable request.
